# Ten-Year Outcomes in Liver Transplantation for HBV/HDV Coinfection: A Romanian Single-Center Cohort Study

**DOI:** 10.3390/jcm15208193

**Published:** 2026-10-21

**Authors:** Speranta Iacob, Mirela Chitul, Vlad Brasoveanu, Razvan Cerban, Ileana Constantinescu, Bogdan Dorobantu, Carmen Ester, Cristian Gheorghe, Mihaela Ghioca, Mugur Grasu, Vlad Herlea, Doina Hrehoret, Razvan Iacob, Ioana Lupescu, Emil Matei, Irinel Popescu, Liana Gheorghe

**Affiliations:** 1 Center for Digestive Diseases and Liver Transplantation, Fundeni Clinical Institute, 022328 Bucharest, Romania; mirela_onica@yahoo.com (M.C.); drvladbrasoveanu@gmail.com (V.B.); cerbanrazvan@yahoo.com (R.C.); ileana.constantinescu@imunogenetica.ro (I.C.); carmen.ghidu@hotmail.com (C.E.); drcgheorghe@gmail.com (C.G.); lita.mihaela.corina@gmail.com (M.G.); cristian.grasu@umfcd.ro (M.G.); herlea2002@yahoo.com (V.H.); doinitahrehoret@yahoo.com (D.H.); raziacob@gmail.com (R.I.); ilupescu@gmail.com (I.L.); emmatei@yahoo.com (E.M.); irinel.popescu220@gmail.com (I.P.); drlgheorghe@gmail.com (L.G.); 2 Faculty of General Medicine, Carol Davila University of Medicine and Pharmacy, 020021 Bucharest, Romania

**Keywords:** hepatitis D virus, hepatitis B virus, liver transplantation, HBsAg recurrence, hepatocellular carcinoma, anti-HBs, hepatitis B immunoglobulin, Romania

## Abstract

**Background/Objectives**: Chronic hepatitis B virus (HBV)/hepatitis D virus (HDV) coinfection is a major cause of cirrhosis and hepatocellular carcinoma (HCC) and remains a disproportionately frequent indication for liver transplantation (LT) in Romania, one of the highest-HDV-prevalence countries in Europe, yet patient-level outcome data from this high-burden Eastern European setting are lacking. We report the ten-year outcomes of LT for HBV/HDV-related cirrhosis in a large single-centre Romanian cohort. **Methods**: We retrospectively analysed 236 adults transplanted for HBV/HDV-related cirrhosis at a single tertiary centre. Survival was estimated by the Kaplan-Meier method and predictors were assessed by Cox regression. HBV, HDV and HCC recurrence, and anti-HBs kinetics under combined hepatitis B immunoglobulin (HBIG)/nucleos(t)ide-analogue prophylaxis, were analysed. **Results**: Over a median follow-up of 8.1 years (maximum 14.4), overall survival was 89.8%, 82.8% and 79.5% at 1, 5 and 10 years; among 90-day survivors, conditional survival reached 86.9% at 10 years. Under an “on-demand” low-dose HBIG protocol combined with nucleos(t)ide analogues, HBV recurrence (HBsAg reappearance) occurred in 4.7% and HDV recurrence in 1.7% of patients, without graft dysfunction or death attributable to viral recurrence; anti-HBs titres stabilised at 120–140 IU/L beyond the first year, with approximately 60% of patients maintaining levels above 100 IU/L long-term. HBsAg reappearance occurred exclusively in HCC recipients (11/87 vs. 0/149, *p* < 0.001) and clustered in patients with tumour recurrence (9/17 vs. 2/70, *p* < 0.001), preceding the radiological diagnosis of recurrence in six of the nine patients concerned. Mortality was concentrated in the perioperative period (90-day mortality 8.5%), driven mainly by sepsis and surgical complications, and recurrent HCC was the most common recorded cause of late death. Neither the severity of liver disease at listing (MELD, ALBI) nor any viral variable was associated with survival; HCC as indication was associated with survival in univariable analysis and beyond-Milan tumour burden independently (HR 2.74, 95% CI 1.36–5.54). **Conclusions**: LT for HBV/HDV-related cirrhosis achieves excellent long-term survival and very low viral recurrence under combined prophylaxis, even in this HDV-hyperendemic setting; beyond the perioperative period, viral factors were not associated with survival, whereas beyond-Milan tumour burden was associated with worse survival in one of the sensitivity analyses. In this HBV/HDV cohort, HBsAg reappearance after transplantation for HCC was associated with tumour recurrence and may be regarded as a warning sign prompting oncological reassessment; whether it represents a true early marker requires prospective validation.

## 1. Introduction

Chronic hepatitis D remains the most severe form of chronic viral hepatitis, depending on the hepatitis B virus (HBV) surface antigen (HBsAg) to complete its life cycle and driving accelerated progression to cirrhosis, hepatic decompensation and hepatocellular carcinoma (HCC) [1]. Whereas chronic hepatitis C is now curable with direct-acting antivirals and chronic hepatitis B can be durably suppressed with nucleos(t)ide analogues, effective treatment for HDV has long remained an unmet need. This gap has only recently begun to narrow with the entry inhibitor bulevirtide, the first agent to show disease-modifying activity, although its long-term effect on hard clinical endpoints is still being established [2]. Although vaccination has reduced the pool of susceptible individuals in high-income countries, the global burden remains substantial, with tens of millions of people affected worldwide [3], and HDV persists or re-emerges in endemic foci and through migration [4]. HDV superinfection leads to cirrhosis in the majority of patients within 5–10 years and carries a markedly increased risk of HCC [1,5].

Romania consistently reports one of the highest HDV prevalences in Europe among HBsAg-positive individuals [6]. Prospective national data confirm a persistently high burden over the past decade [7], with particularly elevated rates in vulnerable communities [8] and HDV is a major driver of HCC among Romanian patients with chronic hepatitis [9]. Consequently, HBV/HDV coinfection remains a disproportionately frequent indication for liver transplantation (LT) in this setting, in contrast to lower-prevalence regions where its share is declining. A poster presented at ESOT 2025 by our group [10] showed that, over the period 2000–2024, HBV- and HCV-related etiologies declined among liver transplant candidates in Romania’s largest transplant program, whereas HBV/HDV coinfection became an increasingly prominent indication for liver transplantation in recent years.

Historically, HBsAg-positive recipients and HDV-coinfected patients in particular were regarded as a high-risk transplant population, because graft reinfection was almost universal before effective prophylaxis and five-year survival was only around 50% [11]. The combination of hepatitis B immunoglobulin (HBIG) and high-genetic-barrier nucleos(t)ide analogues transformed this picture, reducing HBV recurrence to approximately 1% and rendering outcomes comparable or superior to other indications [12,13]. Recent large series have reinforced this view: an Italian national intention-to-treat analysis of 494 coinfected candidates reported better survival than in HBV-monoinfected patients with minimal viral recurrence [14]; a 30-year single-centre Italian experience found similar survival with sepsis as the leading cause of death [15]; in addition, a United States registry study concluded that HDV is not an independent predictor of post-transplant mortality [16,17]. However, existing large datasets are multicentre or registry-based or originate from regions where the infection is uncommon and patient-level data from Eastern Europe are lacking. Romania combines one of the highest HDV prevalence rates in Europe with centralised referral of these patients, allowing the assembly of a uniformly managed cohort with no direct counterpart in the current literature.

We therefore report the ten-year outcomes of 236 patients transplanted for HBV/HDV-related cirrhosis at a single Romanian centre. To our knowledge, this is one of the largest single-centre coinfected LT cohorts reported to date, comparable in size to roughly half of Italy’s entire national multicentre coinfected cohort [14], but accrued and managed within one institution under uniform surgical and prophylactic practice. We focus on survival and its predictors, viral and tumour recurrence under an individualised low-dose HBIG protocol, the dynamics of anti-HBs titres and the relationship between HBsAg reappearance and HCC recurrence.

## 2. Materials and Methods

### 2.1. Study Design and Population

We performed a retrospective analysis of a prospectively maintained institutional database comprising all adult patients who underwent LT for HBV/HDV coinfection-related cirrhosis at a single tertiary transplant centre. A total of 236 consecutive patients transplanted between January 2012 and December 2022 were included; the entire cohort had documented HBV/HDV coinfection, defined as HBsAg positivity with detectable anti-HDV antibodies and documented HDV viraemia at any time before transplantation. Follow-up data were updated until database lock, so that the last patients accrued a minimum of 3.5 years of potential follow-up. The database was locked in June 2026, and all analyses reflect the information available at that date. The study was conducted in accordance with the Declaration of Helsinki and was approved by the Institutional Review Board (approval no. 11176/18.03.2025). Given the retrospective nature of the study and the use of clinical and laboratory data routinely collected as part of standard post-transplant follow-up at predefined intervals, specific informed consent for participation in this study was not required.

### 2.2. Data Collection and Definitions

For each patient we retrieved demographic data, the MELD, MELD-Na and MELD 3.0 scores, the albumin-bilirubin (ALBI) score and complications at listing (variceal bleeding, hepatic encephalopathy, refractory ascites, spontaneous bacterial peritonitis, hepatorenal syndrome and portal vein thrombosis). HCC present as a transplant indication was classified according to the Milan criteria [18], and alpha-fetoprotein (AFP) was recorded at listing. Milan status refers to the last cross-sectional imaging before transplantation, after any bridging or downstaging treatment; explant pathology was used to confirm the diagnosis. Prior antiviral history was recorded, including treatment with pegylated interferon alpha (PEG-IFN) for hepatitis D before decompensation and the use of nucleos(t)ide analogues. No patient received bulevirtide before transplantation, as the drug was not available in Romania during the enrolment period. Pre-transplant virological assessment included quantitative HBsAg, anti-HBs, HBeAg, anti-HBe, HBV DNA, anti-HDV, and HDV RNA. Serological markers were assessed using ELISA-based assays, while HBV DNA and HDV RNA were quantified by real-time PCR and reported as detectable, with viral load expressed in IU/mL, or undetectable. HBV DNA quantification was performed using the Bosphore HBV Quantification Kit (Anatolia Geneworks, Istanbul, Turkey). All 31 patients with undetectable HDV RNA at listing had a documented positive HDV RNA result earlier in their disease course; some had also received PEG-IFN. HDV RNA can fluctuate and may become undetectable during follow-up, including as advanced liver disease evolves or after treatment. An undetectable result at listing therefore does not negate the documented history of infection or imply that liver disease had stopped progressing. Clinical data were collected during hepatology clinic visits and included fibrosis stage, liver stiffness measured by transient elastography, and abdominal ultrasound findings. The MELD-Na score was used as a measure of disease severity at listing [19].

Post-transplant HBV immunoprophylaxis was monitored through serial anti-HBs titres at 1 week and at 1, 3, 6, 12, 24 and 60 months. HBV recurrence was defined as reappearance of serum HBsAg after transplantation, confirmed on a second sample; HBV DNA was quantified at the time of HBsAg reappearance and during subsequent follow-up. HDV recurrence was defined as detectable serum HDV RNA after transplantation, tested in all patients with HBsAg reappearance, and HCC recurrence as radiologically or histologically confirmed tumour relapse. Perioperative (early) mortality was defined as death within 90 days of transplantation.

### 2.3. HBV Immunoprophylaxis

All patients received combined prophylaxis with HBIG and a nucleos(t)ide analogue using an institutional “on-demand” low-dose protocol adapted from our previously reported Romanian transplant experience [20]. HBIG (10,000 IU) was administered intravenously during the anhepatic phase and daily during the first postoperative week, followed by 2500 IU doses given on demand (intravenously or subcutaneously) whenever anti-HBs fell below the target threshold, set at 150–200 IU/L during the first six months after transplantation and at <100 IU/L thereafter. HBIG was not withdrawn in any patient during the study period. Nucleos(t)ide-analogue therapy was started immediately after transplantation and continued indefinitely: lamivudine was used in the early period, and all patients were switched to entecavir or tenofovir as high-genetic-barrier agents became available in the Romanian treatment programme. Anti-HBs titres were monitored at 1 week and at 1, 3, 6, 12, 24 and 60 months, and at every visit thereafter. This individualised, titre-guided approach anticipated the subsequent ELITA recommendations favouring reduced-dose or individualised HBIG regimens in low-risk recipients [12].

### 2.4. Statistical Analysis

Continuous variables are summarised as median (interquartile range, IQR) or mean ± standard deviation (SD), and categorical variables as counts and percentages; denominators reflect the number of patients with available data. Overall survival was measured from transplantation to death from any cause, with patients alive at last contact censored at that date. Survival was estimated by the Kaplan-Meier method with 95% confidence intervals (CI), and median follow-up was computed using the reverse Kaplan-Meier estimator. Subgroups were compared with the log-rank test, and conditional survival was calculated among 90-day survivors. Predictors of survival were assessed using Cox proportional hazards regression. Candidate variables were first examined in univariable analyses. The multivariable model included age, sex, MELD 3.0, and HCC, with the number of covariates limited by the number of deaths. Two additional models assessed the robustness of the findings: one replaced MELD 3.0 with ALBI, and the other replaced HCC with beyond-Milan status. Because the Milan classification applies only to HCC recipients, beyond-Milan status was coded in the whole-cohort models as a binary variable (beyond-Milan HCC versus all other recipients, i.e., HCC within Milan and non-HCC indications combined), so that it contrasts recipients with advanced tumour burden against the remainder of the cohort rather than within-Milan against beyond-Milan HCC; the latter comparison was examined separately in the HCC subgroup by the log-rank test. The association between HBsAg reappearance and HCC status or recurrence was tested with Fisher’s exact test. Hazard ratios (HR) are reported with 95% CI and discrimination as Harrell’s C-index. A two-sided *p* < 0.05 was considered significant.

## 3. Results

### 3.1. Baseline Characteristics

The cohort comprised 236 patients, of whom 149 (63.1%) were male, with a mean age of 56.2 ± 12.1 years. Liver dysfunction at listing was moderate, with a median MELD of 16 (IQR 13–21), MELD-Na 20 (16–25), MELD 3.0 19 (14–24) and ALBI −2.23 (−2.71 to −1.86). Most patients received a whole graft from a deceased donor (213, 90.3%), while 23 (9.7%) received a graft from a living related donor. The most common complications before LT were variceal bleeding (39, 16.5%), portal vein thrombosis (36, 15.3%; none malignant), hepatic encephalopathy (26, 11.0%) and refractory ascites (21, 8.9%). Maintenance immunosuppression was tacrolimus-based in 200 patients (84.7%), sirolimus-based in 25 (10.6%), and cyclosporine-based in the remaining 11 (4.7%).

HCC was a transplant indication in 87 of 236 patients (36.9%). Tumour burden at the last pre-transplant imaging was predominantly favourable: 62/87 (71.3%) were within and 25/87 (28.7%) beyond the Milan criteria. Among HCC recipients, median AFP at listing was 18.0 ng/mL (IQR 6.4–80.0), with 18/81 (22.2%) above 100 ng/mL and 8/81 (9.9%) above 400 ng/mL; in the whole cohort median AFP was 7.4 ng/mL (IQR 3.4–22.1). HDV RNA was undetectable before transplantation in 31 patients (13.1%), while the remaining 205 (86.9%) had detectable viremia (median HDV RNA, 183,600 IU/mL; IQR, 26,175–1,455,000). HBeAg was positive in 34 patients (14.4%). Although HBV DNA had been detectable during the pre-transplant period in 59 patients (25.0%), all received entecavir therapy and achieved undetectable HBV DNA by the time of LT. A history of PEG-IFN therapy for hepatitis D was documented in 53/211 patients (25.1%); none had received bulevirtide. The median interval from HDV diagnosis to transplantation was 4.5 years (IQR 1.7–8.8) and from listing to transplantation 5.2 months (IQR 1.8–11.3). All grafts were from donors after brain death or from living donors.

### 3.2. Dynamics of Anti-HBs Titres

**Figure 1:**
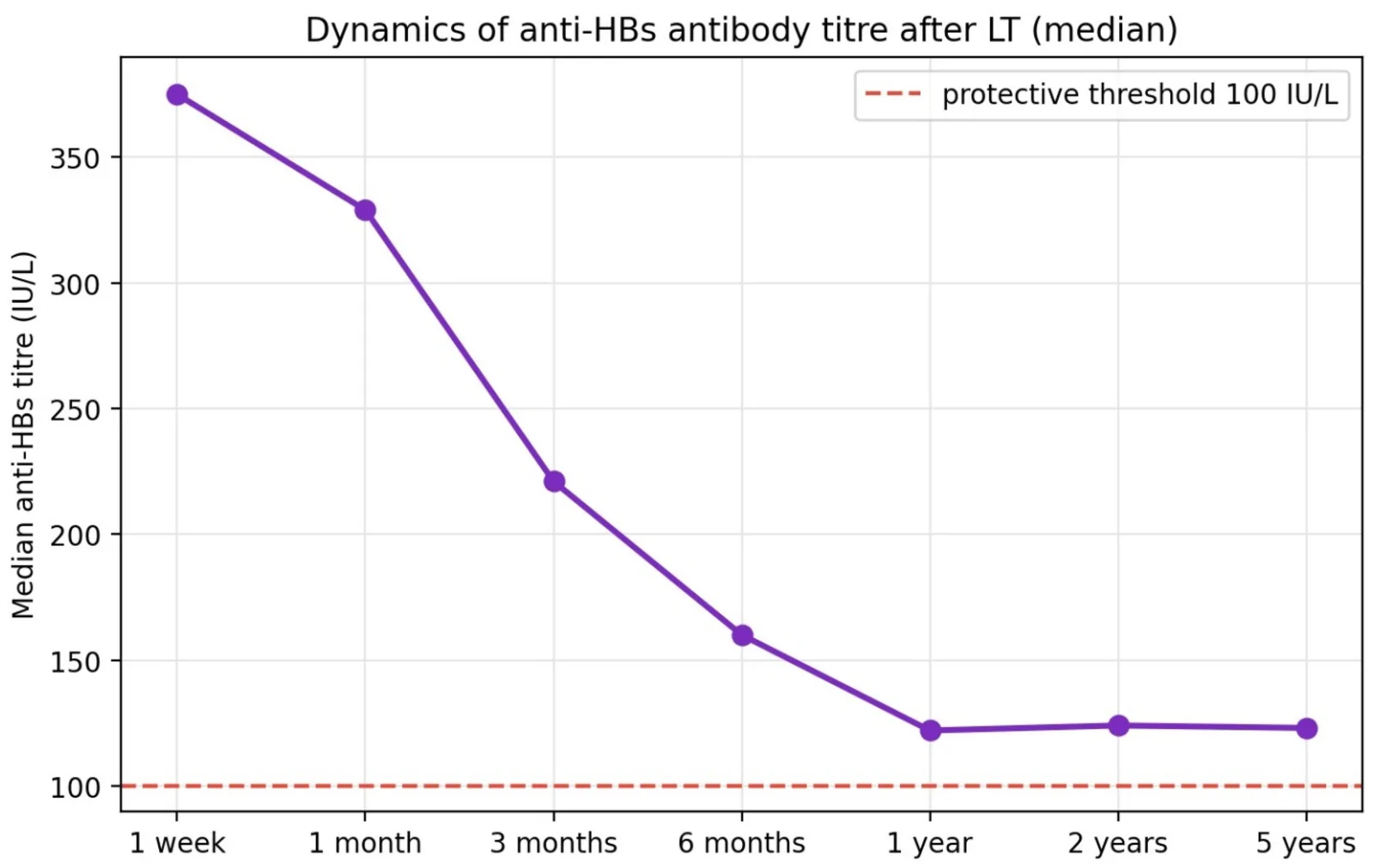
Median anti-HBs titre over time; the dashed line marks the 100 IU/L protective threshold.

Under immunoprophylaxis, anti-HBs titres peaked early (mean ≈ 510 IU/L at 1 week) and declined progressively before stabilising at ≈120–140 IU/L (median) beyond the first year. In the first year the mean titre was ≈141 IU/L (median 122, IQR 83–191), and approximately 60% of patients maintained titres above 100 IU/L in the long term (Table 1, Figure 1).

### 3.3. Post-Transplant Recurrences and the HBV–HCC Relationship

**Figure 2:**
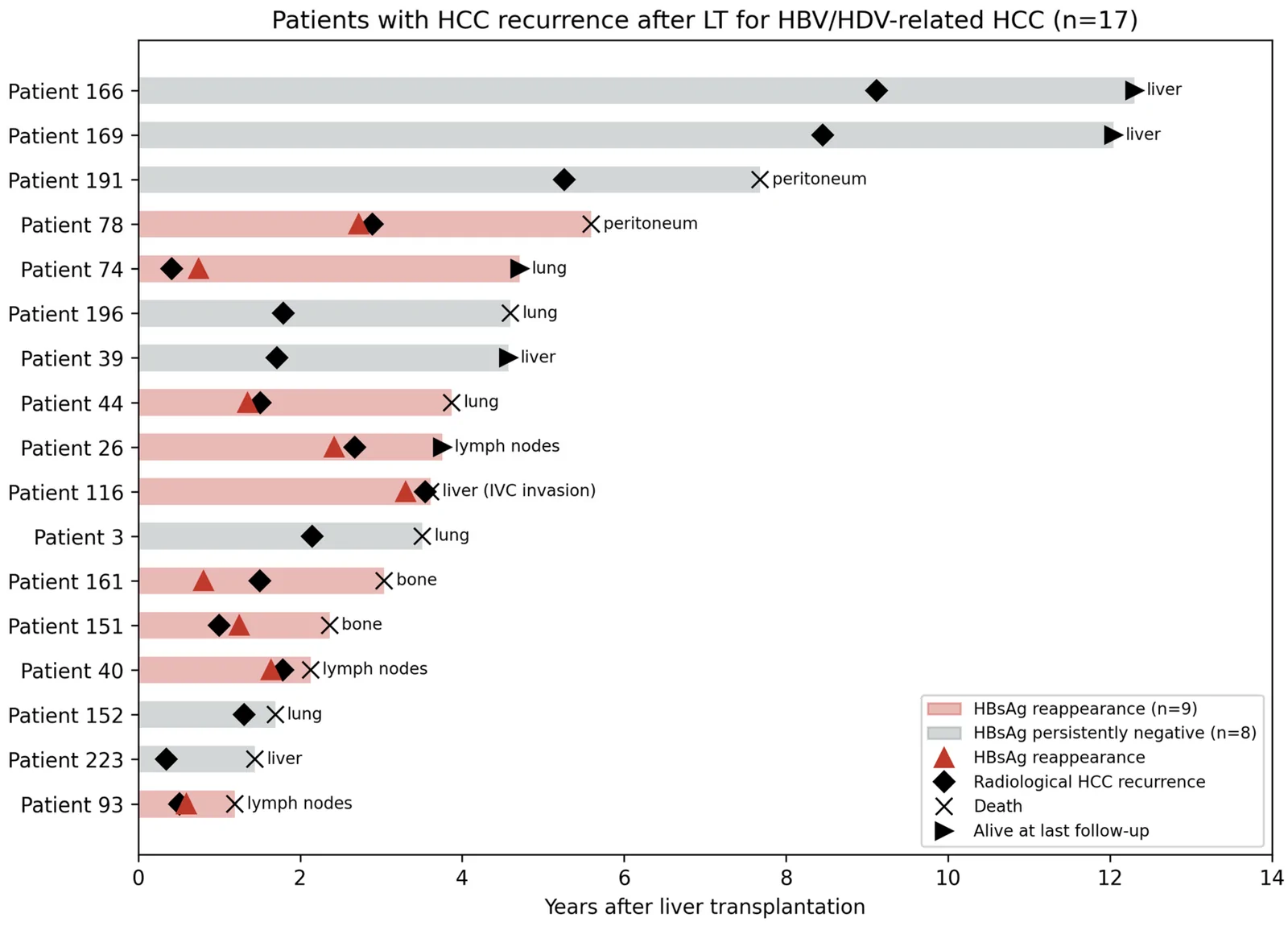
Swimmer plot of the 17 patients with HCC recurrence after transplantation. Red bars, patients with HBsAg reappearance (*n* = 9); grey bars, patients with persistently negative HBsAg (*n* = 8). Triangles mark HBsAg reappearance, diamonds the radiological diagnosis of recurrence, crosses death and arrowheads patients alive at last follow-up.

HBsAg reappeared in 11/236 patients (4.7%), all transplanted for HCC (11/87 versus 0/149 without HCC; *p* < 0.001), at a median of 15 months (range 0.2–40). At reappearance, HBV DNA was detectable in eight patients (13–2133 IU/mL), undetectable in three. Four patients had documented HDV recurrence (1.7%; HDV RNA 1500–14,800,000 IU/mL). None developed graft hepatitis or clinically significant liver dysfunction attributable to viral recurrence. HCC recurred in 17/87 recipients (19.5%). HBsAg reappearance occurred in 9/17 patients with HCC recurrence versus 2/70 without (*p* < 0.001). In six of the nine patients with both events, HBsAg preceded radiological recurrence. Figure 2 shows the individual timelines. Given the small number of events, the possibility that HBsAg reappearance signals HCC recurrence requires prospective validation.

### 3.4. Overall and Conditional Survival

**Figure 3:**
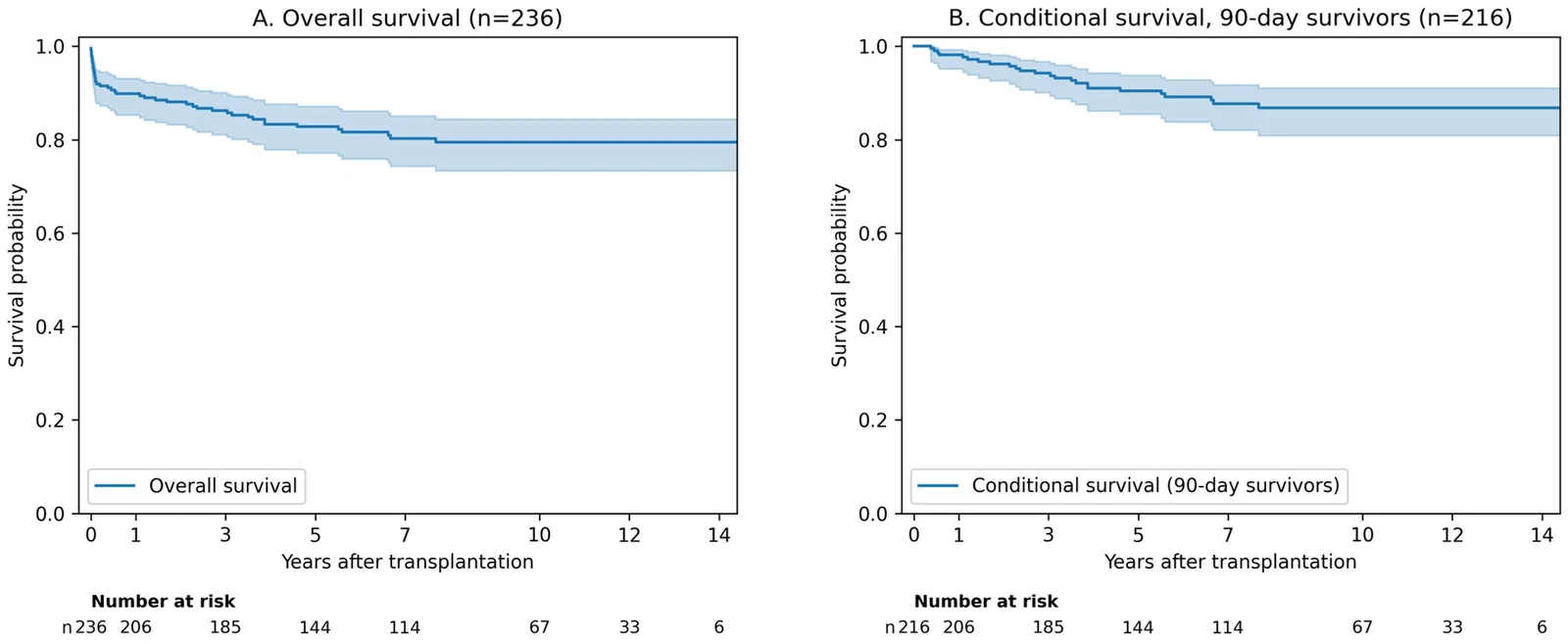
Kaplan–Meier overall survival (**A**) and conditional survival among 90-day survivors (**B**); shaded areas denote 95% confidence intervals and the number of patients at risk is shown below each panel.

Survival was analysed in all 236 patients, among whom 44 deaths occurred over a median follow-up of 8.1 years (IQR 4.7–10.9; maximum 14.4 years). Overall survival was 89.8% (95% CI 85.2–93.1) at 1 year, 86.2% (81.1–90.1) at 3 years, 82.8% (77.2–87.1) at 5 years and 79.5% (73.3–84.4) at 10 years. Twenty of the 44 deaths (45.5%) occurred within 90 days (90-day mortality 8.5%), mainly from sepsis, haemoperitoneum/haemorrhagic shock and primary graft non-function. Among the 216 patients still under observation at day 90, conditional survival was 98.1%, 94.2%, 90.4% and 86.9% at 1, 3, 5 and 10 years (Table 2, Figure 3).

### 3.5. Causes of Death and Predictors of Survival

**Figure 4:**
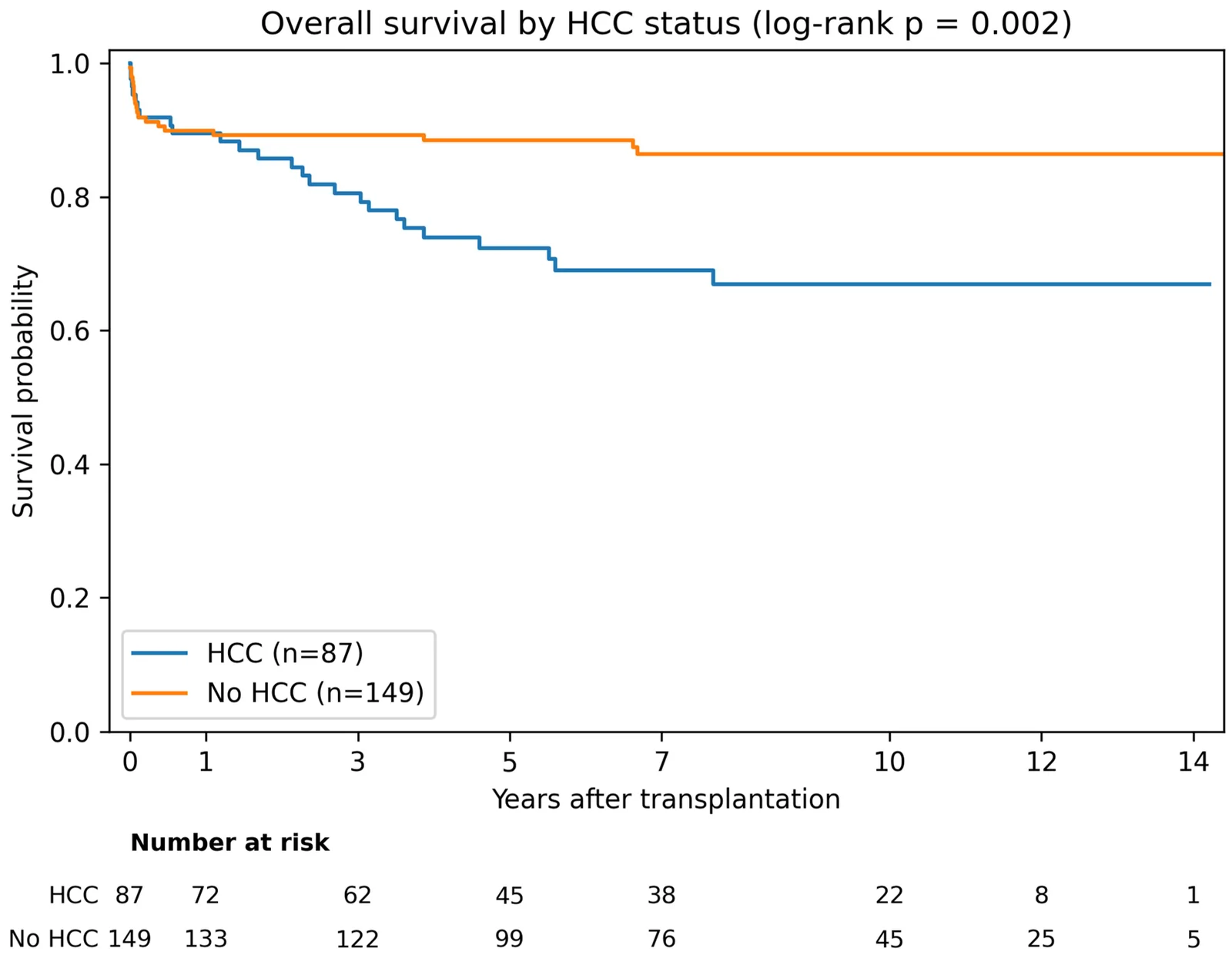
Kaplan–Meier overall survival according to HCC as transplant indication (log-rank *p* = 0.002); the number of patients at risk is shown below the plot.

Of the 44 deaths, 20 occurred within and 24 beyond 90 days. Early deaths were due to sepsis (n = 6), haemoperitoneum or haemorrhagic shock (n = 5), primary graft non-function or hepatic artery thrombosis (n = 3) and intracranial haemorrhage (n = 2), with the cause undocumented in four. The most common recorded causes of late death were recurrent HCC (n = 10) and sepsis (n = 6), the remainder being cardiovascular, de novo malignancy or other causes; no death was attributable to HBV or HDV recurrence per se, and the seven deaths among the 11 patients with HBsAg reappearance were all due to tumour progression. In univariable Cox regression (Table 3), neither MELD, MELD 3.0, ALBI, the pre-transplant complications of cirrhosis, the interval from HDV diagnosis to transplantation nor the waiting time was associated with survival; older age was associated with mortality (HR 1.03 per year, 95% CI 1.00–1.06, *p* = 0.031), as were HCC as indication (HR 2.42, 1.33–4.39, *p* = 0.004) and beyond-Milan tumour burden (HR 3.08, 1.55–6.10, *p* = 0.001); 5- and 10-year survival were 72.6% and 67.2% in HCC recipients versus 88.5% and 86.4% in patients without HCC (log-rank *p* = 0.002; Figure 4), and 5-year survival was 77.3% within versus 61.2% beyond Milan (*p* = 0.066). In the multivariable model (age, sex, MELD 3.0, HCC; 211 patients, 43 events; C-index 0.60) neither age nor HCC reached significance (HCC: HR 1.83, 0.95–3.53, *p* = 0.071), whereas in the sensitivity model in which HCC was replaced by beyond-Milan status (coded as beyond-Milan HCC versus all other recipients), beyond-Milan tumour burden was associated with mortality (HR 2.74, 1.36–5.54, *p* = 0.005); replacing MELD 3.0 with ALBI did not change these results. Within the HCC subgroup, the difference between within- and beyond-Milan recipients did not reach statistical significance.

## 4. Discussion

In this large single-centre Romanian cohort, LT for HBV/HDV-related cirrhosis produced excellent long-term results, with 5- and 10-year survival of 82.8% and 79.5% and very low rates of viral recurrence under an individualised low-dose HBIG protocol. Several clinically relevant observations emerged from our analysis. Mortality was concentrated in the perioperative period, mainly because of sepsis and surgical complications, whereas neither liver disease severity at listing nor the measured viral characteristics were associated with survival. Beyond-Milan tumour burden was associated with mortality in one of the sensitivity analyses, although the within- versus beyond-Milan comparison in the HCC subgroup did not reach significance. Anti-HBs protection remained durable under immunoprophylaxis, supporting the sustained effectiveness of the preventive strategy. Finally, HBsAg reappearance occurred only in HCC recipients and was closely associated with tumour recurrence, confirming in an HBV/HDV-coinfected cohort an association already described in HBV-monoinfected recipients.

Our survival figures are concordant with the best contemporary series, but derive from a distinctive source. The Italian national intention-to-treat analysis reported 5-year post-transplant survival of 83.2% in coinfected patients, better than in HBV-monoinfected candidates, yet that cohort of 494 patients was assembled across 22 transplant centres and included waitlist follow-up [14]. A 30-year single-centre Italian experience reported comparable survival (5-year 82.6% overall and 83.7% in the HDV subgroup), although only around one-third of its 321 HBsAg-positive recipients were HDV-coinfected [15], and a US registry study confirmed that HDV does not independently worsen post-transplant survival [16]. With 236 coinfected recipients transplanted and followed under a consistent institutional protocol, this series adds one of the largest single-centre HBV/HDV experiences reported to date and, importantly, provides data from a hyperendemic Eastern European setting that the transplant literature has largely overlooked. HBV and HDV recurrence occurred in 4.7% and 1.7% of our patients, respectively. These rates can be compared with contemporary cohorts under combined HBIG and nucleos(t)ide-analogue prophylaxis, in which HBV recurrence is often around or below 1% and HDV recurrence is uncommon [13,14]; in the Italian national series HBV and HDV recurrences were 1.1% and 0.2% [14], and no HDV-coinfected recipient experienced HBV recurrence in the Padua cohort [15]. The higher HBV recurrence in our series may reflect the proportion of HCC recipients, the length of follow-up or differences in prophylaxis. It should also be noted that our protocol deliberately maintained lower anti-HBs targets than those used in most contemporary series, with HBIG given on demand rather than at fixed intervals [20]; HBV DNA at the time of HBsAg reappearance was low or undetectable in most cases, and no recurrence resulted in graft dysfunction or death attributable to viral infection. Our serial anti-HBs measurements offer a clearer view of the long-term antibody response: titres rose early and then stabilised at approximately 120–140 IU/L, while around 60% of patients remained above the conventional 100 IU/L threshold during follow-up. Whereas early protocols targeted anti-HBs above 500 IU/L, subsequent work showed that levels above 100 IU/L suffice to prevent graft reinfection [13]. In the Italian national cohort, 8.2% of patients discontinued HBIG without recurrence, and those maintaining anti-HBs above 100 IU/L appeared to do so safely [14]; a Spanish multicentre study reported that more than 40% of HDV-related recipients stopped HBIG one to two years after LT with an HDV recurrence rate of only 1.7% [21]. Although guidelines still recommend indefinite dual prophylaxis [22,23], the trend toward HBIG-sparing or high-barrier NA monotherapy in low-risk recipients is growing [23,24,25,26] and our anti-HBs measurements describe the titres achieved with continued, individualised HBIG dosing. The increased risk of HBV recurrence in liver transplant recipients with HCC has been recognised for more than fifteen years: HBV recurrence was four times more frequent in HCC than in non-HCC recipients in a Turkish series, where 7 of 8 tumour recurrences were accompanied by HBV recurrence [27]; in the UCLA experience, HCC before transplantation and tumour recurrence after it were the only independent predictors of HBV reinfection [28]; in a recent Chinese two-centre study, HBV reactivation after transplantation for HCC was associated with tumour recurrence and metastasis [29]; and the Italian nationwide analysis confirmed that HBV recurrence, although rare (3.1% in HCC recipients), was independently associated with HCC recurrence without affecting survival [30]. In our cohort, HBsAg reappearance was confined to HCC recipients and was more frequent in those with than without tumour recurrence. One proposed explanation is that extrahepatic tumour cells containing integrated HBV DNA may act as a reservoir that is not fully suppressed by nucleos(t)ide analogue therapy [13]. This possibility is supported by the Italian national series, in which the only episode of HBV recurrence among patients with HCC occurred one year after tumour relapse [14]. Both HCC and HDV coinfection are therefore considered settings in which closer post-transplant monitoring may be warranted [13].

Our data are consistent with this association and, with the limitation of a small number of events, provide some information on its timing. In six of the nine patients with both events, HBsAg became detectable before tumour relapse was identified radiologically, mainly with extrahepatic recurrence (lymph nodes, lung, bone or peritoneum), sites that may be difficult to recognise at an early stage on routine cross-sectional imaging. A similar sequence was reported in a Taiwanese series, in which HBV recurrence preceded HCC recurrence in 7 of 10 recipients, by one to fifteen months [31]. In a region where HDV remains an important contributor to HCC risk [9], the reappearance of HBsAg in a recipient with HBV/HDV coinfection transplanted for HCC may therefore deserve attention not only as a virological event, but also as a warning sign prompting oncological reassessment; the available data do not allow it to be defined as a true early marker of recurrence. The reported persistence of intrahepatic HBV markers and hepatitis delta antigen after transplantation, despite apparent serological control, provides a biologically plausible explanation for this observation [32,33]. These findings must, however, be interpreted cautiously. Only 11 HBsAg reappearance events occurred, two of them in patients without tumour recurrence to date, and the apparent association with HCC recurrence is therefore based on a small number of patients. Although the sequence was consistent in six of the nine patients concerned, the retrospective nature of the study does not allow us to determine whether HBsAg reappearance is directly related to tumour burden or whether it simply identifies patients with a disease phenotype associated with both virological recurrence and tumour relapse. We also cannot establish whether earlier imaging or any other intervention triggered by isolated HBsAg reappearance would have changed clinical management or outcome. This observation should therefore be regarded as hypothesis-generating and requires prospective validation through predefined serological monitoring combined with structured oncological imaging in recipients transplanted for HCC.

Although HDV recurrence was rare in our cohort and did not cause graft dysfunction, its management after transplantation deserves comment because therapeutic options remain limited. Reinforcement of prophylaxis (HBIG re-administration and a high-genetic-barrier nucleos(t)ide analogue) is the first step, since suppression of HBsAg deprives HDV of its envelope. PEG-IFN, the only historical option, is poorly tolerated and carries a risk of acute rejection in transplant recipients, and is therefore not used [22]. Bulevirtide, an entry inhibitor approved in Europe in 2020, has been used safely in patients awaiting transplantation, where it improved liver function and facilitated HCC treatment [34], but experience after transplantation is limited to case reports [35]. Bulevirtide has a very low drug–drug interaction potential, although monitoring of tacrolimus levels is advised during co-administration. Whether bulevirtide has a role in the treatment of post-transplant HDV recurrence, or in the pre-transplant reduction of the HDV reservoir, remains to be established prospectively [2,22]. In our four patients with HDV recurrence, all of whom had HBsAg reappearance, management consisted of reinforcement of HBIG and nucleos(t)ide-analogue prophylaxis; none developed graft hepatitis.

The pattern of mortality observed in our cohort also deserves comment. Almost half of all deaths occurred within 90 days of transplantation and were related to sepsis and surgical complications, a pattern also reported in the Padua cohort, where sepsis was the leading cause of death both overall and among recipients with HDV infection [15]. Beyond this period, recurrent HCC was the most common recorded cause of late death, and among baseline variables HCC as indication was associated with survival in univariable analysis but not in the multivariable model, whereas beyond-Milan status was associated with worse survival in one of the sensitivity analyses; given the small number of events and the non-significant within- versus beyond-Milan comparison in the HCC subgroup, this finding should be interpreted with caution. Neither the severity of liver disease at listing (MELD, MELD 3.0, ALBI), the pre-transplant complications of cirrhosis, the documented interval from HDV diagnosis to transplantation nor any measured viral characteristic was associated with post-transplant survival. Because some patients were tested for HDV only after referral, this documented interval may underestimate the duration of HDV infection. In the Italian national intention-to-treat analysis, HCC, older age and higher MELD-Na were associated with mortality largely because of their effect on waitlist outcomes [14]; our analysis included only patients who underwent transplantation and therefore did not capture mortality or disease progression before transplant. Taken together, these results indicate that further improvements in outcome may depend as much on perioperative care, the prevention and management of infectious complications and the selection and surveillance of HCC recipients as on the optimisation of antiviral prophylaxis, in line with current guideline recommendations [22,23,36]. Several limitations should be acknowledged. This was a retrospective, single-centre study, which may limit the generalisability of the findings and does not allow complete control of potential unmeasured confounding. The number of HBsAg reappearance events and deaths was small, reducing statistical power and limiting the robustness of multivariable analyses. Donor- and graft-related variables were not available, so the determinants of early mortality could not be analysed. The cohort predates pre-transplant bulevirtide use in our centre. The effect of this treatment on transplant and post-transplant outcomes in currently treated candidates warrants prospective evaluation. The study was also restricted to transplanted patients and did not include waitlisted patients who died or were delisted, preventing an intention-to-treat assessment. Furthermore, the retrospective design precluded a fully standardised prospective assessment of all study variables and outcomes.

The study also has several strengths. It includes a relatively large cohort of liver transplant recipients with HBV/HDV infection from an HDV-hyperendemic region that remains underrepresented in the transplant literature, where survival outcomes have historically been reported at a broader European level [37]. Follow-up extended to 10 years, allowing assessment of both early and late outcomes. Serial anti-HBs measurements provided a detailed longitudinal view of post-transplant antibody kinetics, while management within a single centre ensured a broadly consistent clinical approach. Nevertheless, these strengths do not remove the need for external and prospective validation of the observed associations.

## 5. Conclusions

Liver transplantation for HBV/HDV-related cirrhosis in this Romanian HDV-endemic setting was associated with excellent long-term survival and a very low rate of HBV recurrence under combined, individualised low-dose prophylaxis, markedly lower than rates reported in the early post-transplant era [38]. The sustained anti-HBs response observed during follow-up supports the feasibility of individualised long-term prophylactic strategies. Post-transplant mortality was concentrated in the early perioperative period, largely because of infectious and surgical complications, whereas the severity of liver disease and viral factors were not associated with survival; beyond-Milan status was associated with worse survival in one of the sensitivity analyses. HBsAg reappearance in recipients transplanted for HCC was associated with tumour recurrence, confirming in this HBV/HDV cohort a previously described association; it may be regarded as a warning sign prompting oncological reassessment, although its value as an early marker of recurrence requires prospective validation. As one of the larger single-centre cohorts of HBV/HDV-coinfected liver transplant recipients reported to date, this study provides further evidence that transplantation offers excellent long-term outcomes in this population, including in a high-prevalence Eastern European setting.

## Figures and Tables

**Table 1:** Anti-HBs titres (IU/L) after transplantation.

Time Point	Mean	Median (IQR)	>100 IU/L
1 week	510	376 (92–764)	73%
1 month	394	330 (200–490)	90%
3 months	265	222 (158–317)	89%
6 months	201	160 (105–241)	76%
1 year	141	122 (83–191)	61%
2 years	131	124 (76–194)	62%
5 years	138	123 (72–176)	61%

**Table 2:** Overall and conditional (90-day survivors) survival, with the number of patients at risk at each time point.

Time from LT (n at Risk)	Overall Survival (95% CI)	Conditional Survival (95% CI)
30 days (222)	93.6% (89.6–96.1)	—
90 days (216)	91.5% (87.1–94.4)	—
1 year (206)	89.8% (85.2–93.1)	98.1% (95.1–99.3)
3 years (185)	86.2% (81.1–90.1)	94.2% (90.0–96.7)
5 years (144)	82.8% (77.2–87.1)	90.4% (85.3–93.8)
10 years (67)	79.5% (73.3–84.4)	86.9% (80.8–91.1)

**Table 3:** Univariable Cox regression for overall survival.

Variable	HR (95% CI)	*p*
Age (per year)	1.03 (1.00–1.06)	0.031
Female sex	1.06 (0.58–1.94)	0.848
MELD	0.99 (0.94–1.04)	0.576
MELD 3.0	1.00 (0.96–1.04)	0.949
ALBI score	1.14 (0.74–1.75)	0.554
HCC (indication)	2.42 (1.33–4.39)	0.004
Beyond Milan	3.08 (1.55–6.10)	0.001
Portal vein thrombosis	1.47 (0.69–3.17)	0.321
Variceal bleeding	1.51 (0.73–3.16)	0.270
HDV diagnosis to LT (per year)	0.98 (0.92–1.04)	0.519
Waiting time (per month)	0.99 (0.97–1.01)	0.384

## Data Availability

The data presented in this study are available from the corresponding author on reasonable request.

## Abbreviations

The following abbreviations are used in this manuscript:

**Abbreviation**	**Definition**
HBV	Hepatitis B virus
HDV	Hepatitis D virus
HCC	Hepatocellular carcinoma
LT	Liver transplantation
HBsAg	Hepatitis B surface antigen
HBIG	Hepatitis B immunoglobulin
MELD	Model for End-Stage Liver Disease
ALBI	Albumin-Bilirubin (score)
AFP	Alpha-fetoprotein
IQR	Interquartile range
CI	Confidence interval
HR	Hazard ratio
SD	Standard deviation
PCR	Polymerase chain reaction
DCD	Donation after circulatory death
PEG-IFN	Pegylated interferon alpha
